# Immune-related adverse events and neutrophil-to-lymphocyte ratio as prognostic indicators in gynecologic cancer patients receiving pembrolizumab: a real-world analysis

**DOI:** 10.3389/fimmu.2025.1739447

**Published:** 2026-01-21

**Authors:** Chien-Hsiang Kao, Hao Lin, Yu-Che Ou, Hung-Chun Fu, Ching-Chou Tsai, Chen-Hsuan Wu

**Affiliations:** 1 Department of Obstetrics and Gynecology, Kaohsiung Chang Gung Memorial Hospital and Chang Gung University College of Medicine, Kaohsiung, Taiwan; 2 Graduate Institute of Clinical Medical Sciences, College of Medicine, Chang Gung University, Taoyuan, Taiwan

**Keywords:** biomarkers, gynecologic malignancies, immune checkpoint inhibitors, immune-related adverse events, immunotherapy, mismatch repair deficiency, neutrophil-to-lymphocyte ratio, pembrolizumab

## Abstract

**Objective:**

To investigate whether immune-related adverse events and pretreatment neutrophil-to-lymphocyte ratio can serve as predictive biomarkers of treatment response and survival in patients with gynecologic cancer receiving pembrolizumab immunotherapy.

**Methods:**

This retrospective study included 94 patients with gynecologic malignancies treated with pembrolizumab at Kaohsiung Chang Gung Memorial Hospital between May 2017 and July 2024. Detailed clinical and laboratory data including the occurrence of immune-related adverse events, pretreatment neutrophil-to-lymphocyte ratio, mismatch repair (MMR) status, treatment response patterns, and patient demographics were collected. Progression-free survival and overall survival were analyzed using Kaplan-Meier curves and Cox regression models. The optimal neutrophil-to-lymphocyte ratio cut-off value for predicting the prognosis was determined using receiver operating characteristic curve analysis.

**Results:**

Overall, immune-related adverse events occurred in 55.3% of the patients and were associated with a significantly higher objective response rate (ORR; 84.6% vs. 15.4%, *p* < 0.001), longer progression-free survival (*p* < 0.001), and improved overall survival (*p* < 0.001). Similarly, low neutrophil-to-lymphocyte ratio (<4.07) also predicted longer progression-free survival (HR: 0.537, *p* = 0.043) and improved overall survival (HR: 0.328, *p* = 0.001). Multivariate analysis confirmed that both immune-related adverse events and low neutrophil-to-lymphocyte ratio were robust independent predictors of progression-free survival and overall survival. MMR-deficient tumors were associated with a significantly higher ORR (60.9% vs. 28.9%, *p* = 0.011), although MMR status was not independently associated with survival in the final multivariate models.

**Conclusion:**

The development of immune-related adverse events and low pretreatment neutrophil-to-lymphocyte ratio independently predicted improved therapeutic response and prolonged survival in gynecologic cancer patients treated with pembrolizumab. Their integration with tumor molecular profiling may optimize monitoring frequency, adjust supportive care measures, and consider treatment modifications based on individual patient risk profiles, thereby enhancing the delivery of immunotherapy in gynecologic cancers without limiting treatment access.

## 1 Introduction

Over the past decade, immunotherapy has transformed cancer treatment, markedly enhancing the prognosis of patients with various types of cancer​ (1, 2). In particular, immune checkpoint inhibitors (ICIs) including cytotoxic T-lymphocyte-associated protein 4 (CTLA-4), programmed cell death protein 1 (PD-1), and programmed death-ligand 1 (PD-L1) have shown promise in treating gynecological malignancies (3, 4). Several therapeutic strategies have emerged that integrate ICIs with conventional treatment to enhance response rates and prolong survival. For example, integrating pembrolizumab with chemotherapy has been shown to improve outcomes in patients with advanced endometrial cancer, particularly for tumors with deficient mismatch repair (dMMR) (5). Similarly, combining pembrolizumab with chemoradiotherapy has shown encouraging results in the treatment of locally advanced cervical cancer (6). However, the increased use of new immunotherapy combinations has also raised safety concerns, particularly regarding immune-related adverse events.

Immune-related adverse events are caused by immune activation induced by ICIs, and they most commonly affect the skin, gastrointestinal tract, and endocrine system (7). In our clinical experience, gynecologic cancer patients who develop immune-related adverse events often have improved responses to immunotherapy. However, while growing evidence from other cancer types suggests a positive association between immune-related adverse events and treatment outcomes, data on gynecologic malignancies remain limited (8, 9). Given the increasing use of immunotherapy, further investigations are warranted to determine whether immune-related adverse events may serve as predictive markers of treatment efficacy.

Beyond immune-related adverse events, the identification of reliable biomarkers to predict the response to immunotherapy remains a critical issue due to the heterogeneity in clinical outcomes. Established biomarkers such as PD-L1 expression, MMR, microsatellite instability, and tumor mutational burden can offer valuable insights into patient selection (10, 11). However, a substantial proportion of patients fail to achieve a durable response, underscoring the need for additional biomarkers to optimize treatment outcomes. Recent efforts have focused on simple blood-based indices as potential predictive markers, and particularly the neutrophil-to-lymphocyte ratio (12). The neutrophil-to-lymphocyte ratio reflects the systemic inflammatory response and can serve as a surrogate marker of the balance between pro-tumor and anti-tumor immune activity. An elevated baseline neutrophil-to-lymphocyte ratio has been associated with a poor prognosis in various solid tumors, and recent studies have suggested that it may also be correlated with the outcomes of immunotherapy, including response and survival (13). Despite its potential, the role of neutrophil-to-lymphocyte ratio remains underexplored in gynecologic malignancies.

As the interplay between neutrophil-to-lymphocyte ratio, immune-related adverse events, and treatment efficacy remains unclear, this study aimed to evaluate whether the development of immune-related adverse events and pretreatment neutrophil-to-lymphocyte ratio could serve as predictive markers of treatment response and survival in gynecologic cancer patients receiving pembrolizumab.

## 2 Materials and methods

This study retrospectively analyzed a consecutive cohort of patients with gynecologic malignancies who received pembrolizumab at Kaohsiung Chang Gung Memorial Hospital between May 2017 and July 2024. Patients were eligible for inclusion if they had a pathologically confirmed diagnosis of gynecological malignancy and had received at least one cycle of pembrolizumab. Patients were excluded if they had synchronous cancers, previously received immunotherapy, not been treated or followed up at the hospital, or incomplete records. The primary objective of this study was to evaluate differences in objective response rate (ORR), progression-free survival and overall survival based on the occurrence of immune-related adverse events. This study was conducted in compliance with relevant ethical guidelines and approved by the Institutional Review Board of Chang Gung Memorial Hospital (No. 202402017B0).

Demographic and clinical variables included age at diagnosis, body mass index (BMI), Eastern Cooperative Oncology Group Performance Status (ECOG-PS), cancer type, International Federation of Gynecology and Obstetrics (FIGO) stage at diagnosis, and number of prior lines of chemotherapy. The neutrophil-to-lymphocyte ratio was recorded immediately preceding the first pembrolizumab administration. Tumor MMR status was assessed using immunohistochemical analysis of MLH1, MSH2, MSH6, and PMS2 expressions. dMMR was defined as the loss of expression of at least one of these proteins. Immunotherapy-related data included pembrolizumab dose per cycle, total number of cycles, combination treatment regimen, and the interval to immune-related adverse event onset. Immune-related adverse events (irAEs) were defined as adverse events with suspected immune-mediated etiology occurring after pembrolizumab therapy onset, for which alternative etiologies (e.g., infection, disease progression, or toxicity from concomitant treatments) were not considered more likely based on clinical evaluation and available diagnostic work-up. Potential irAEs were identified via systematic review of electronic medical records, including oncology progress notes, subspecialty consultation notes, laboratory results, and imaging reports. Events were reviewed and confirmed as irAEs when documented as immune-mediated and managed accordingly by the treating gynecologic oncologist and/or relevant subspecialists. All irAEs were graded using the Common Terminology Criteria for Adverse Events (CTCAE) version 5.0 and categorized by organ system (endocrine, gastrointestinal, hepatic, pulmonary, dermatologic, renal, neurologic, and others). If multiple irAEs occurred in a patient, the earliest event was used to define time-to-onset, and the highest CTCAE grade was used to summarize severity at the patient level. Clinical data were retrieved from electronic medical records.

Progression-free survival was defined as the interval from the start of pembrolizumab to disease progression or recurrence. Overall survival was defined as the time to death from any cause or last follow-up. Objective tumor response was evaluated using the Response Evaluation Criteria in Solid Tumors (RECIST 1.1), and the ORR was defined as the percentage of patients with a complete response or partial response. Pretreatment NLR was available for all patients because complete blood counts were routinely obtained prior to pembrolizumab administration as part of standard care. Response assessment was performed according to RECIST v1.1; patients without adequate post-baseline imaging or clinical evidence to evaluate the response were considered not evaluable for response and were excluded from response-rate calculations, with denominators reported for each analysis, and categorized as “Could not evaluate”.

Continuous variables were reported as means and ranges, and categorical variables as numbers and percentages. Survival was analyzed using the Kaplan-Meier method, with group differences assessed by the log-rank test. The optimal pretreatment neutrophil-to-lymphocyte ratio cut-off was determined via receiver operating characteristic curve analysis. The threshold was selected by maximizing the Youden index (sensitivity + specificity − 1) to identify the cut-off that best balanced sensitivity and specificity. Univariate and multivariate Cox proportional hazards models identified significant and independent prognostic predictors of treatment response and survival, respectively. Statistical analyses were conducted using SPSS 25.0 (IBM, Armonk, NY, USA), with a two-sided p-value <0.05 considered significant.

In accordance with the journal’s guidelines, we will provide our data for independent analysis by a selected team by the Editorial Team for the purposes of additional data analysis or for the reproducibility of this study in other centers if such is requested.

## 3 Results

This study enrolled 94 gynecologic cancer patients with a median age of 55.5 years (range, 32–83), 11.7% classified as being older (≥70 years). Most (66.0%) had an ECOG-PS score of 0, with diagnoses of corpus uteri (46.8%), cervical (33.0%), ovarian (13.8%), or vaginal cancer (6.4%). FIGO stage III (29.8%) or IV (45.7%) was prevalent at diagnosis. Pembrolizumab was used as first-line (7.5%), second line (34.0%), or third-line/later therapy (58.5%), with a mean pretreatment neutrophil-to-lymphocyte ratio of 4.2 (range, 1.4–92.0). Of 71 patients with MMR testing, 33.8% were MMR-deficient; among 20 cervical cancer patients with PD-L1 testing, 55.0% had a combined positive score (CPS) ≥1. Combination therapy was used in 61.7% of patients, with 39.4% (n=37) receiving pembrolizumab plus chemotherapy and 34.0% (n=32) receiving pembrolizumab with anti-angiogenic agents. The median number of pembrolizumab cycles was 8.7 (range, 1–64). Most patients (88.3%, n=83) received a 100 mg fixed dose per cycle (**Table 1**).

**Table 1:** Baseline demographic and clinical characteristics of the included patients (n = 94).

Characteristics	Number of patients, n (%)
Age, years	
Median (range)	55.5 (32-83)
Older (≥ 70)	11 (11.7)
BMI, median, kg/m^2^ (range)	23.7 (15.3-37.5)
Median follow-up time, month (IQR)	9.6 (4.3-15.2)
ECOG PS	
0	62 (66.0)
1	29 (30.9)
2	3 (3.1)
Cancer type	
Endometrium	44 (46.8)
Cervix	31 (33.0)
Ovary/Fallopian	3 (13.8)
Vagina	6 (6.4)
FIGO stage at diagnosis	
I	12 (12.8)
II	11 (11.7)
III	28 (29.8)
IV	43 (45.7)
Timing of pembrolizumab	
First line	7 (7.5)
Second line	32 (34.0)
Third or more	55 (58.5)
Combination therapy	
ICI only	36 (38.3
Chemotherapy^a^	37 (39.4
Anti-angiogenic agents^b^	32 (34.0)
Number of ICI cycles	
Median (IQR)	5.5 (2.75-12)
Dose (mg)	
50	2 (2.1)
100	83 (88.3)
200	9 (9.6)
Best treatment response	
Complete response	15 (16.0)
Partial response	24 (25.5)
Stable disease (SD)	7 (7.4)
Progressive disease (PD)	44 (46.8)
Could not evaluate	4 (4.3)
Median time to response, month (range)	2.9 (1.6-5.6)
MMR status	
Deficient	24 (25.5)
Proficient	47 (50.0)
Unknown	23 (24.5)
Neutrophil-to-lymphocyte ratio, median (range)	4.2 (1.4-92.0)
Median time to immune-related adverse event, days (range)^c^	89.5 (5-546)

^a^Any combination of pembrolizumab with chemotherapy.

^b^Any combination of pembrolizumab with anti-angiogenic agents, including bevacizumab (anti-VEGF monoclonal antibody) and lenvatinib (VEGFR-targeted tyrosine kinase inhibitor).

Overall, immune-related adverse events of any grade were observed in 52 patients (55.3%) with median time to onset was 89.5 days (range, 5 to 546) (**Supplementary Table 1**). Among them, 46 had low-grade immune-related adverse events, including 28 with grade I and 18 with grade II, while 6 had grade III immune-related adverse events. No grade IV immune-related adverse events or fatal adverse events were reported. The most frequently observed immune-related adverse event was skin related (46.1%), followed by diarrhea (26.9%), hypothyroidism (23.1%) and hyperthyroidism (11.5%). The occurrence of immune-related adverse events was significantly associated with treatment response (**Table 2**). Patients who developed immune-related adverse events achieved a markedly higher ORR compared to those without immune-related adverse events (84.6% vs. 15.4%, p < 0.001). However, when stratified by immune-related adverse event type, neither skin-related nor thyroid-related immune-related adverse events were significantly associated with treatment response (**Supplementary Table 2**). The development of immune-related adverse events was not influenced by pembrolizumab dose (p = 0.290) or MMR status (p = 0.153). The pretreatment NLR cut-off was selected based on ROC curve analysis by identifying the threshold that maximized discrimination. The patients with low pretreatment neutrophil-to-lymphocyte ratio (<4.62) demonstrated a significantly higher incidence of irAE than those with high neutrophil-to-lymphocyte ratio (68.6% vs. 39.5%, p = 0.005). Additionally, patients with low pretreatment neutrophil-to-lymphocyte ratio (<4.54) demonstrated a significantly higher ORR than those with high neutrophil-to-lymphocyte ratio (56.0% vs. 27.5%, p = 0.007).

**Table 2:** Correlations between immune-related adverse events, treatment response, and clinical factors.

Clinical factors	Immune-related adverse events	n (%)	No immune-related adverse events	n (%)	P value	Complete response + partial response	n (%)	SD + PD	n (%)	P value
Age (years)					0.210					0.177
< 70	48	(57.8)	35	(42.2)		37	(46.3)	43	(53.7)	
≥ 70	4	(36.4)	7	(63.6)		2	(20.0)	8	(80.0)	
BMI (kg/m^2^)					0.887					0.598
< 24	28	(56.0)	22	(44.0)		20	40.8)	29	(59.2)	
≥ 24	24	(54.5)	20	(45.5)		19	(46.3)	22	(53.7)	
ECOG PS					0.105					0.015
0	38	(61.3)	24	(38.7)		31	(52.5)	28	(47.5)	
≥ 1	14	(43.8)	18	(56.3)		8	(25.8)	23	(74.2)	
FIGO stage at diagnosis					0.205					0.528
I	5	(41.7)	7	(58.3)		4	(36.4)	7	(63.6)	
II	5	(45.5)	6	(54.5)		5	(55.6)	4	(44.4)	
III	20	(71.4)	8	(28.6)		14	(51.9)	13	(48.1)	
IV	22	(51.2)	21	(48.8)		16	(37.2)	27	(62.8)	
Timing of pembrolizumab					0.216					0.011
1st line	6	(85.7)	1	(14.3)		6	(85.7)	1	(14.3)	
2nd line	18	(56.3)	14	(43.7)		16	(53.3)	14	(46.7)	
3rd line or more	28	(50.9)	27	(49.1)		17	(32.1)	36	(67.9)	
Combination therapy					0.042					0.042
ICI only	14	(38.9)	22	(61.1)		7	(21.2)	26	(78.8)	
Chemotherapy	15	(57.7)	11	(42.3)		15	(57.7)	11	(42.3)	
Anti-angiogenic agents	14	(66.7)	7	(33.3)		9	(42.9)	12	(57.1)	
Chemotherapy + anti-angiogenic agents	9	(81.8)	2	(18.2)		8	(80.0)	2	(20.0)	
Dose					0.290					0.290
Low dose^a^	49	(57.6)	36	(42.4)		37	(45.7)	44	(54.3)	
Regular dose^b^	3	(33.3)	6	(66.7)		2	(22.2)	7	(77.8)	
MMR status (n=71)					0.153					0.011
Deficient	17	(70.8)	7	(29.2)		14	(60.9)	9	(39.1)	
Proficient	25	(53.2)	22	(46.8)		13	(28.9)	32	(71.1)	
Neutrophil-to-lymphocyte ratio					0.005					0.007
< 4.62	35	(68.6)	16	(31.4)						
≥ 4.62	17	(39.5)	26	(60.5)						
< 4.54						28	(56.0)	22	(44.0)	
≥ 4.54						11	(27.5)	29	(72.5)	
Immune-related adverse events					NA					1.000
Low grade	NA	NA	NA	NA		29	(63.0)	17	(37.0)	
High grade	NA	NA	NA	NA		4	(66.7)	2	(33.3)	
Best treatment response					<0.001					NA
Complete response + Partial response	33	(84.6)	6	(15.4)		NA	NA	NA	NA	
SD + PD	19	(37.3)	32	(62.7)		NA	NA	NA	NA	

Bold values denote statistical significance (*p* < 0.05).

^a^ Low dose was defined as a dosage of less than 200 mg per treatment cycle.

^b^ Regular dose was defined as a fixed dose of 200 mg per treatment cycle.

At the time of data cut-off, the median follow-up duration was 9.6 months (range, 0.4 to 89.8). A total of 39 patients had an objective response (ORR = 41.5%), including 15 with a complete response (38.5%) and 24 with a partial response (61.5%). In addition, 44 patients had progressive disease, while the treatment response could not be evaluated in 4 patients. ECOG-PS and combination therapy were significantly correlated with an objective response (*p* < 0.05). Moreover, dMMR status was significantly associated with a higher response rate (60.9% vs. 28.9%, *p* = 0.011). In contrast, age, BMI, FIGO stage at diagnosis, pembrolizumab dose per cycle, and immune-related adverse event grade were not significantly correlated with ORR (**Table 2**).

**Figure 1:**
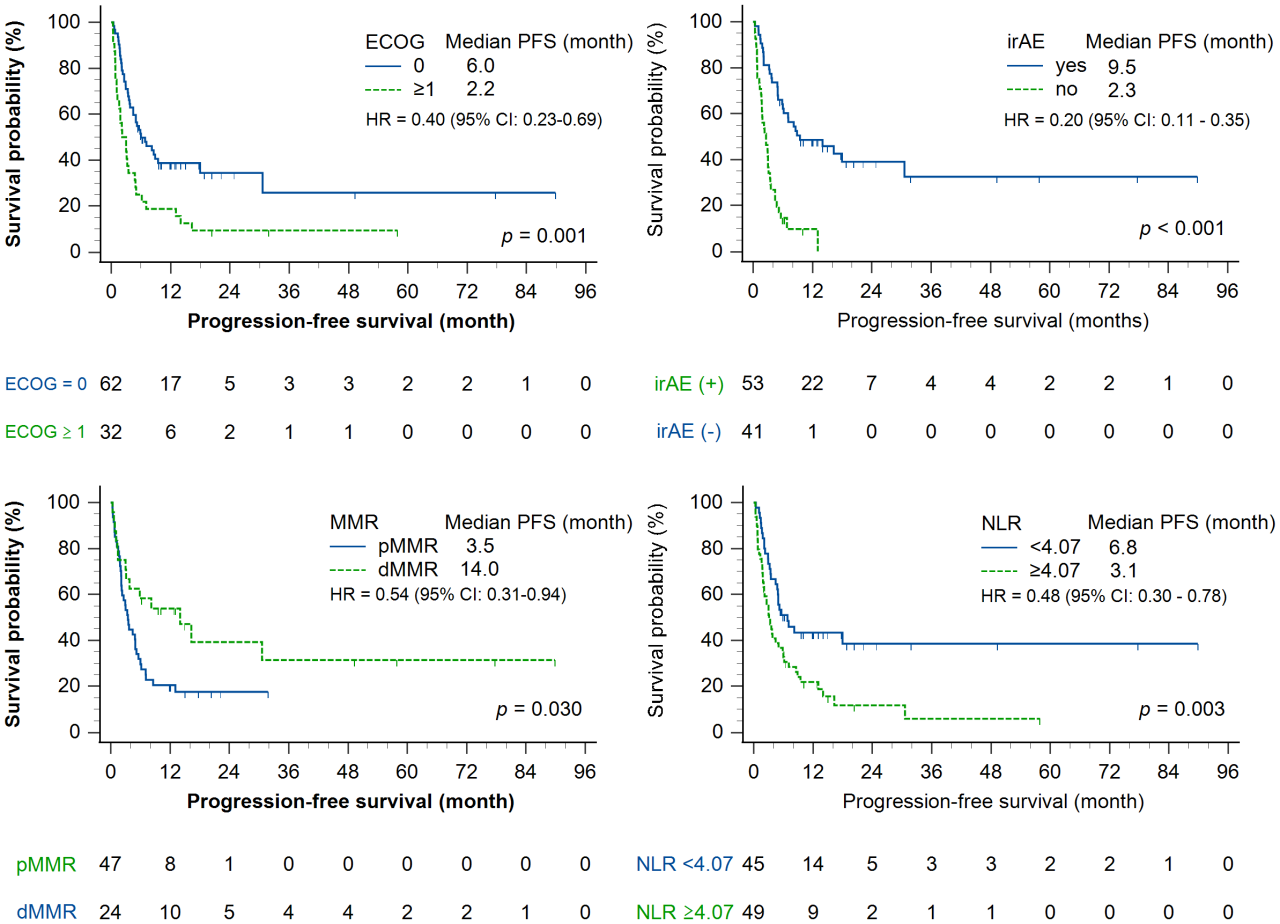
Kaplan–Meier curves for progression-free survival stratified by clinical and biological factors. Progression-free survival in patients treated with pembrolizumab, stratified by (upper left) ECOG PS (0 vs. ≥1), (upper right) presence or absence of immune-related adverse events, (lower left) MMR status (deficient vs. proficient), and (lower right) pretreatment neutrophil-to-lymphocyte ratio (<4.07 vs. ≥4.07). Statistically significant differences in progression-free survival were observed for ECOG, immune-related adverse events, and neutrophil-to-lymphocyte ratio groups (log-rank *p* < 0.05).

**Figure 2:**
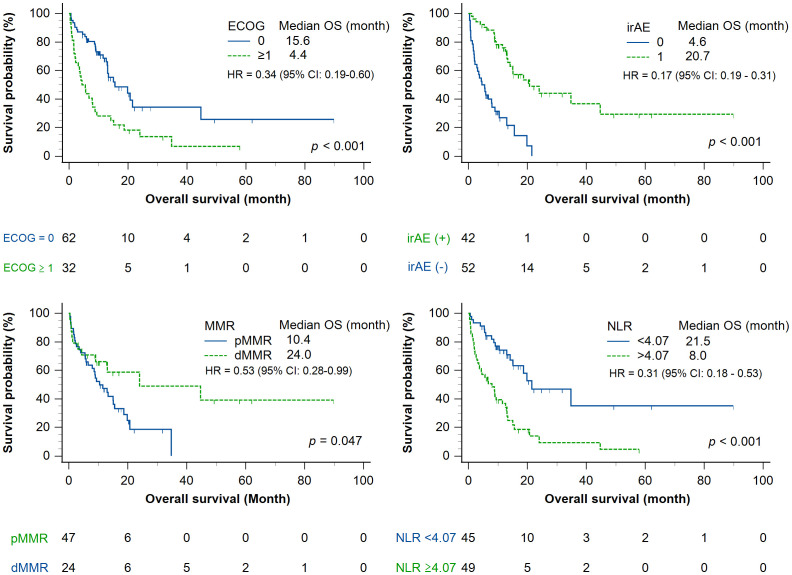
Kaplan–Meier curves for overall survival stratified by clinical and biological factors. Overall survival in patients treated with pembrolizumab, stratified by (upper left) ECOG PS (0 vs. ≥1), (upper right) presence or absence of immune-related adverse events, (lower left) MMR status (deficient vs. proficient), and (lower right) pretreatment neutrophil-to-lymphocyte ratio (<4.07 vs. ≥4.07). Patients with ECOG = 0, immune-related adverse event development, dMMR, or low neutrophil-to-lymphocyte ratio exhibited significantly prolonged overall survival. Statistical comparisons were performed using the log-rank test.

**Figure 3:**
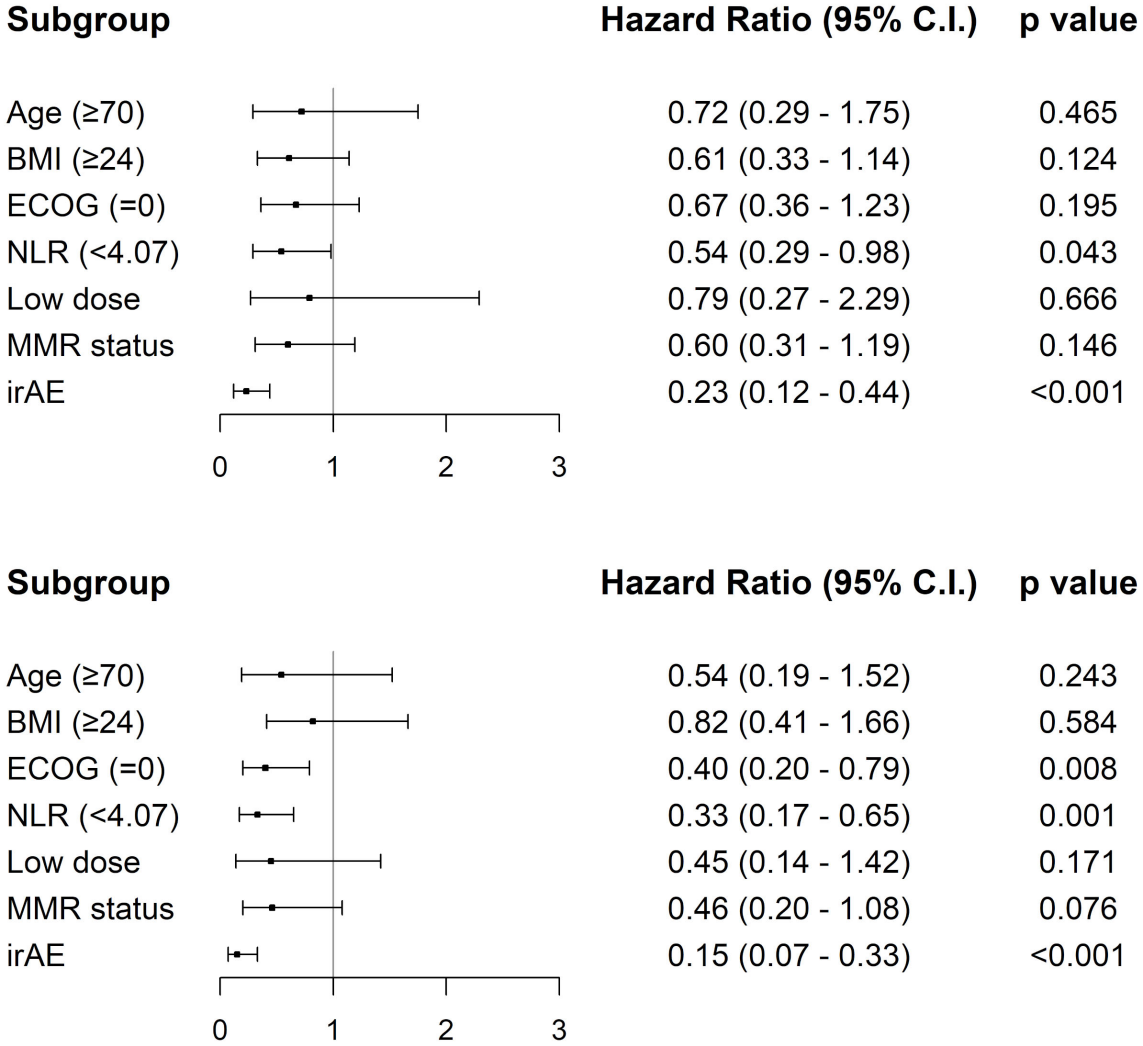
Forest plots of multivariate analysis for progression-free survival and overall survival. Multivariate Cox proportional hazards models for (top) progression-free survival and (bottom) overall survival in patients receiving pembrolizumab. HRs and 95% CI are shown for each subgroup. Lower pretreatment neutrophil-to-lymphocyte ratio (<4.07) and the occurrence of immune-related adverse events were independently associated with improved progression-free survival and overall survival. Additionally, an ECOG PS score of 0 was significantly associated with better overall survival. Statistical significance was defined as *p* < 0.05.

In the univariate analysis, a lower neutrophil-to-lymphocyte ratio (<4.07) and ECOG-PS score of 0 were significantly associated with longer progression-free survival (*p* = 0.003 and *p* = 0.001, respectively). The occurrence of an immune-related adverse event (*p* < 0.001) and dMMR status (*p* = 0.030) were also associated with prolonged progression-free survival (**Figure 1**). In contrast, age and pembrolizumab dose were not significantly associated with progression-free survival. Regarding overall survival, neutrophil-to-lymphocyte ratio, ECOG-PS, occurrence of an immune-related adverse event, and MMR status acted as significant prognostic factors in univariate analysis (**Figure 2**), while age and treatment dose were not. In the multivariate analysis, both low neutrophil-to-lymphocyte ratio and the occurrence of an immune-related adverse event remained independent predictors of longer progression-free survival, with hazard ratios (HRs) of 0.54 (95% CI, 0.294–0.982; *p* = 0.043) and 0.23 (95% CI, 0.120–0.443; *p* < 0.001), respectively. In contrast, age (≥70 years), BMI (≥24 kg/m^2^), pembrolizumab dose, ECOG-PS, and MMR status were not independently associated with progression-free survival in the multivariate analysis. For overall survival, both low neutrophil-to-lymphocyte ratio (<4.07) and the occurrence of an immune-related adverse event remained independently associated with improved overall survival (HR, 0.33; 95% CI, 0.166–0.650; *p* = 0.001; and HR, 0.15; 95% CI, 0.070–0.330; *p* < 0.001, respectively). Similarly, an ECOG-PS score of 0 was also independently associated with longer overall survival (HR, 0.403; 95% CI: 0.201–0.792; *p* = 0.008), while age, BMI, pembrolizumab dose, and MMR status were not significantly associated with overall survival (**Figure 3**).

## 4 Discussion

### 4.1 Summary of main results

In a real-world study of 94 gynecologic cancer patients treated with pembrolizumab, immune-related adverse events (occurring in 55.3%) were linked to a higher ORR (84.6% vs. 15.4%, p<0.001) and improved progression-free survival and overall survival. Low pretreatment neutrophil-to-lymphocyte ratio (<4.07) independently predicted better progression-free survival (HR: 0.48, p=0.003) and overall survival (HR: 0.31, p<0.001), and was associated with increased immune-related adverse event incidence at a cut-off of 4.62 (p=0.005). Patients with dMMR had higher response rates (60.9% vs. 28.9%, p=0.011), but immune-related adverse events and neutrophil-to-lymphocyte ratio were stronger prognostic factors than MMR status. Integrating immune-related adverse events, neutrophil-to-lymphocyte ratio, and MMR status enhanced prognostic accuracy for treatment response and survival. This biomarker framework supports risk stratification and personalized monitoring during pembrolizumab therapy.

### 4.2 Results in the context of published literature

In this study, immune-related adverse events occurred in 55.3% of the patients, which is comparable to previous reports on the use of pembrolizumab in patients with gynecologic cancers (14, 15). Importantly, the occurrence of immune-related adverse events was strongly associated with better ORR, progression-free survival, and overall survival, corroborating prior evidence that immune-related adverse events may serve as biomarkers of effective immune activation (16–18). The underlying mechanisms may involve bystander immune effects and enhanced T cell reactivity induced by immunotherapy (19). For example, Berner et al. showed that T cells triggered by anti–PD-1 therapy could attack both tumor and healthy skin due to shared antigens​ in patients with non-small cell lung cancer, possibly explaining why skin immune-related adverse events may be associated with tumor regression. This antigenic overlap not only triggers adverse cutaneous effects but also correlates with tumor regression, highlighting the dual impact of T cell-mediated immunity on both therapeutic outcomes and immune-related adverse events (20). Similarly, in melanoma patients receiving ICIs, higher pretreatment levels of activated CD4 effector memory T cells and greater T cell receptor clonotype diversity have been strongly linked to severe immune-related adverse events and improved clinical outcomes (21). This aligns with observations in melanoma and lung cancer that robust immune activation is associated with better tumor control​. The present study extends this concept to gynecologic cancers, for which data were previously limited.​ Our findings demonstrated that the patients who developed immune-related adverse events had a nearly six-fold higher response rate than those without immune-related adverse events, supporting the idea that strong T cell activation drives both treatment efficacy and development of immune-related adverse events.

Building on these findings regarding immune-related adverse events and immune activation, the neutrophil-to-lymphocyte ratio has emerged as another valuable biomarker for predicting response and survival in various malignancies, including non-small cell lung cancer and melanoma (22, 23). The mechanism has been proposed to involve both systemic inflammation and immune status of the tumor microenvironment. Motomura et al. demonstrated that preoperative neutrophil-to-lymphocyte ratio was an independent predictor of hepatocellular carcinoma recurrence after living-donor liver transplantation, driven by an inflammatory tumor microenvironment characterized by elevated IL-17 and CD163+ tumor-associated macrophages (24). Similarly, Takakura et al. reported that an elevated neutrophil-to-lymphocyte ratio was correlated with increased CD163+ macrophages and decreased CD20+ lymphocytes in patients with pancreatic cancer, further supporting the role of neutrophil-to-lymphocyte ratio as a marker of an immunosuppressive tumor microenvironment (25). An elevated neutrophil-to-lymphocyte ratio indicates intensified neutrophilic inflammation and diminished lymphocyte-driven immunity. The immune imbalance contributes to an immunosuppressive tumor microenvironment, potentially undermining the effectiveness of ICIs (26, 27). The pretreatment neutrophil-to-lymphocyte ratio cut-off value for predicting survival benefits from ICIs varies across different cancers, ranging from 0.7 to 11.7 (13, 28). In our study, a pretreatment neutrophil-to-lymphocyte ratio cut-off value of 4.07 was significantly associated with improved progression-free survival and overall survival and demonstrated potential as a clinical prognostic marker.

Both low neutrophil-to-lymphocyte ratio and the occurrence of immune-related adverse events stem from related immunological mechanisms, driven by dysregulated immune responses and increased adaptive immunity. ICIs amplify T cell activity by inhibiting the PD-1/PD-L1 and CTLA-4 pathways, which not only enhances antitumor effects but also increases the risk of autoimmune reactions leading to immune-related adverse events (12). Similarly, a low neutrophil-to-lymphocyte ratio indicates a higher proportion of lymphocytes, indicating increased T cell activity and a predisposition to autoimmunity (29). Previous studies have reported that patients with a low neutrophil-to-lymphocyte ratio are more likely to develop immune-related adverse events during ICI therapy, with cut-off values ranging from 2.3 to 8.58 (30–32). Our results demonstrated a significant association between lower neutrophil-to-lymphocyte ratio and higher immune-related adverse event incidence, suggesting a link between systemic immune activation and treatment-related toxicity. This highlights neutrophil-to-lymphocyte ratio ‘s value as a simple, cost-effective biomarker for immune-related adverse event risk stratification in gynecological cancer patients receiving ICIs. Clinically, incorporating neutrophil-to-lymphocyte ratio into routine assessment could enable early identification of high-risk patients, facilitating personalized monitoring and timely immune-related adverse event management. To our knowledge, this is the first study to evaluate both neutrophil-to-lymphocyte ratio and immune-related adverse events as concurrent predictors of therapeutic response in gynecological cancer patients receiving ICI therapy, addressing a significant knowledge gap beyond conventional dMMR-based prognostic approaches.

### 4.3 Strengths and weaknesses

This study’s strength is its real-world analysis of pembrolizumab in gynecologic cancer patients, integrating immune-related adverse events, ECOG, and neutrophil-to-lymphocyte ratio in a multivariate model to identify reliable predictors of treatment efficacy. However, its retrospective design, limited sample size and heterogenicity of gynecologic cancers limit subgroup analyses of combination treatments, specific immune-related adverse event types, or cancer-specific outcomes. Therefore, our findings should be interpreted as hypothesis-generating and require validation in larger, tumor-specific cohorts. Incomplete MMR and PD-L1 testing may introduce bias, and longitudinal neutrophil-to-lymphocyte ratio changes, disease burden, prior treatment intensity, combination regimens, steroid use were not assessed. Because ECOG PS 3–4 patients were not represented in this cohort, the generalizability of our findings to patients with poor performance status (ECOG PS 3–4) is uncertain and warrants confirmation in larger datasets. Additional inflammation- and nutrition-related biomarkers (e.g., albumin, the platelet-to-lymphocyte ratio, the systemic immune-inflammation index, and the prognostic inflammatory value) were not consistently available at standardized pretreatment time points and were not analyzed; these markers should be evaluated in future prospective or systematically curated real-world studies.

### 4.4 Implications for practice and future research

Our findings build on prior research, such as Ethier et al., who linked elevated neutrophil-to-lymphocyte ratio to worse overall survival (HR 1.57) and event-free survival (HR 1.46) in gynecologic cancers treated with chemotherapy (33). More recently, Huepenbecker et al.’s comprehensive real-world study of immunotherapy in endometrial cancer, notable for its large cohort, evaluated MMR status but did not include neutrophil-to-lymphocyte ratio analysis (34). Pembrolizumab is FDA-approved for microsatellite instability-high (MSI-H)/dMMR solid tumors, with KEYNOTE-158 reporting a 57.1% ORR for endometrial cancer (35). Our study confirms higher ORR in dMMR patients (60.9% vs. 28.9% in pMMR, p=0.011) but shows some dMMR patients still fail to respond. We uniquely identify low neutrophil-to-lymphocyte ratio (<4.62) as a predictor of increased immune-related adverse event incidence in pembrolizumab-treated gynecologic cancers. Multivariate analysis reveals immune-related adverse events, neutrophil-to-lymphocyte ratio, and MMR status as independent prognostic factors, offering a comprehensive framework that outperforms single-biomarker approaches. This integration enables risk-stratified monitoring, with high neutrophil-to-lymphocyte ratio or absent immune-related adverse events prompting more frequent assessments and earlier treatment adjustments, optimizing immunotherapy without limiting access. Prospective, multi-center studies are needed to validate these findings.

## 5 Conclusion

This study is the largest real-world analysis of pembrolizumab outcomes and immune-related adverse events in gynecologic cancer patients. It highlights MMR status, neutrophil-to-lymphocyte ratio, and immune-related adverse events as key predictors of treatment response and survival. Integrating these biomarkers provides a prognostic framework for risk stratification and personalized monitoring during PD-1 blockade therapy. Easily monitored via routine toxicity assessments (immune-related adverse event) and blood tests (neutrophil-to-lymphocyte ratio), these factors guide tailored management strategies. This approach optimizes immunotherapy delivery without restricting access.

## Data Availability

The raw data supporting the conclusions of this article will be made available by the authors, without undue reservation.
