# Supplementary material for: Immune-related adverse events and neutrophil-to-lymphocyte ratio as prognostic indicators in gynecologic cancer patients receiving pembrolizumab: a real-world analysis
Source: Front Immunol. 2026 Jan 21;16:1739447. doi: 10.3389/fimmu.2025.1739447 (PMC12867807; doi:10.3389/fimmu.2025.1739447)
Supplement: Supplementary file 1 [file Table1.docx]

Supplementary Material

# Supplementary Table 1 Association between specific irAE characteristics and treatment response to pembrolizumab

|  | Any grade, n (%) | Grade 3-4, n (%) |
| --- | --- | --- |
| Any adverse event | **52 (55.3)** | **7 (7.5)** |
| Cutaneous | **24 (25.5)** | **3 (3.2)** |
| Colitis/Diarrhea | **16 (17.0)** | **0** |
| Hypothyroidism | **12 (12.8)** | **0** |
| Hyperthyroidism | **6 (6.4)** | **0** |
| Thyroiditis | **2 (2.1)** | **1 (1.1)** |
| Hepatitis | **3 (3.2)** | **0** |
| Myalgia | **2 (2.1)** | **0** |
| Pneumonitis | **2 (2.1)** | **1 (1.1)** |
| Adrenal insufficiency | **1 (1.1)** | **0** |
| Conjunctivitis | **1 (1.1)** | **0** |
| Uveitis | **1 (1.1)** | **1 (1.1)** |
| Periodontitis | **1 (1.1)** | **0** |
| Mucositis | **1 (1.1)** | **0** |
| Nephritis | **1 (1.1)** | **0** |
| Others^a^ | **5 (5.3)** | **1 (1.1)** |

^a^ Others include cases of adrenal insufficiency, herpes zoster, cystitis, hemophagocytic lymphohistiocytosis (HLH), and tinnitus.

# Supplementary Table 2 Association Between Specific Immune-Related Adverse Events Characteristics and Treatment Response to Pembrolizumab

| Immune-related adverse Event | SD + PD | Complete response + Partial response | P value |
| --- | --- | --- | --- |
| Skin-related immune-related adverse event |  |  | 0.924 |
| Yes | 23 (63.9) | 13 (36.1) |  |
| No | 10 (62.5) | 6 (37.5) |  |
| Thyroid-related immune-related adverse event |  |  | 0.741 |
| Yes | 24 (64.9) | 13 (35.1) |  |
| No | 9 (60.0) | 6 (40.0) |  |

The table summarizes the relationship between skin-related irAEs and thyroid-related irAEs with treatment response status (SD+PD vs. CR+PR) in patients treated with pembrolizumab.

irAE, immune-related adverse event; SD, stable disease; PD, progressive disease; CR, complete response; PR, partial response; ICI, immune checkpoint inhibitor.

# Supplementary Table 3 Detailed Chemotherapy Treatment Regimens Used in Combination with Pembrolizumab

| Treatment class | Drug components | Schedule | No. of patients |
| --- | --- | --- | --- |
| Chemotherapy (platinum doublet) | Carboplatin + Paclitaxel + | q3w | n = 25 |
|  | Cisplatin + Paclitaxel | q3w | n = 2 |
|  | Carboplatin + PLD | q3w | n = 1 |
| Chemotherapy (non-platinum) | Paclitaxel | weekly | n = 5 |
|  | PLD | q4w | n = 1 |
|  | Topotecan | q3w | n = 1 |
|  | Gemcitabine | q3w | n = 1 |
|  | Topotecan + paclitaxel | q3w | n = 1 |

PLD, pegylated liposomal doxorubicin; q3w, every 3 weeks; q4w, every 4 weeks.
